# Electrokinetic characterization of synthetic protein nanoparticles

**DOI:** 10.3762/bjnano.11.138

**Published:** 2020-10-13

**Authors:** Daniel F Quevedo, Cody J Lentz, Adriana Coll de Peña, Yazmin Hernandez, Nahal Habibi, Rikako Miki, Joerg Lahann, Blanca H Lapizco-Encinas

**Affiliations:** 1 Biointerfaces Institute, University of Michigan - Ann Arbor, Ann Arbor MI, USA; 2 Biomedical Engineering, University of Michigan - Ann Arbor, Ann Arbor MI, USA; 3 Microscale Bioseparations Laboratory and Biomedical Engineering Department, Rochester Institute of Technology, Rochester NY, USA; 4 Chemical Engineering, University of Michigan - Ann Arbor, Ann Arbor MI, USA

**Keywords:** anisotropy, bicompartmental particles, dielectrophoresis, electrokinetics, electrophoresis, electro-osmosis, microfluidics, protein nanoparticles

## Abstract

The application of nanoparticle in medicine is promising for the treatment of a wide variety of diseases. However, the slow progress in the field has resulted in relatively few therapies being translated into the clinic. Anisotropic synthetic protein nanoparticles (ASPNPs) show potential as a next-generation drug-delivery technology, due to their biocompatibility, biodegradability, and functionality. Even though ASPNPs have the potential to be used in a variety of applications, such as in the treatment of glioblastoma, there is currently no high-throughput technology for the processing of these particles. Insulator-based electrokinetics employ microfluidics devices that rely on electrokinetic principles to manipulate micro- and nanoparticles. These miniaturized devices can selectively trap and enrich nanoparticles based on their material characteristics, and subsequently release them, which allows for particle sorting and processing. In this study, we use insulator-based electrokinetic (EK) microdevices to characterize ASPNPs. We found that anisotropy strongly influences electrokinetic particle behavior by comparing compositionally identical anisotropic and non-anisotropic SPNPs. Additionally, we were able to estimate the empirical electrokinetic equilibrium parameter (e*E*_EEC_) for all SPNPs. This particle-dependent parameter can allow for the design of various separation and purification processes. These results show how promising the insulator-based EK microdevices are for the analysis and purification of clinically relevant SPNPs.

## Introduction

Over the past 30 years, nanoparticles have been developed for a wide variety of scientific applications, ranging from medical imaging to drug delivery and enzyme immobilization to industrial processes [1–2]. The development of nanoparticles composed primarily of proteins has been an emerging sector in nanotechnology [3]. Proteins have multiple desirable characteristics that enable them to be used as the main component of nanoparticles. Proteins are biodegradable, naturally involved in biological molecule targeting, and are “smart” materials that can respond to various environmental cues, such as pH value, temperature, or target binding [4]. Protein nanoparticles (PNPs) are useful for the loading of active therapeutic enzymes and show potential results to be used as vaccines [5–6]. Significant advances in self-assembled PNPs via protein engineering techniques have also been observed [7–8]. PNPs have successfully reached the clinic with nab-paclitaxel (Abraxane^®^), a PNP made of human serum albumin, which is being used for the treatment of metastatic breast cancer, non-small cell lung cancer, and pancreatic adenocarcinomas [9]. Current technologies allow for the synthesis of smart PNPs that release their active enzymatic load into oxidative environments [6]. A next step to further advance smart protein nanoparticle technologies is to develop a scalable method for producing subcompartments within particles. By localizing proteins at the nanoscale, PNPs could be used to control the release and delivery of therapeutics, theragnostics, and enzymatic cascades [10].

Although previously developed methods to produce PNPs, such as coacervation, self-assembly, and pressure-driven techniques, allow for intraparticle spatial control of the material composition through layer-by-layer techniques, no previous synthetic schemes were able to synthesize anisotropic protein nanoparticles with distinct hemispheres [5,11–12]. To address this issue, we have adapted electrohydrodynamic (EHD) co-jetting techniques, previously established by the Lahann lab, to create single-compartment and anisotropic (i.e., multicompartmental) synthetic protein nanoparticles (SPNPs and ASPNPs, respectively), which can be easily made from a variety of proteins [13–15]. A recent publication demonstrated how this versatile technique can be used to create particles with a significant therapeutic potential, in particular to treat glioblastoma [16]. To translate the promising benefits of these particles into the clinic, specific characterization techniques for anisotropic particles need to be developed.

In the last decade, the area of microfluidics, which is the field of science that studies the manipulation of minute volumes of fluids (i.e., from microliters to picoliters) [17], has experienced a significant growth in bioanalytical applications [17–18]. Electrokinetics (EK) and electric-field driven processes are suitable for a wide range of applications due to their simplicity and robustness. An applied electric potential can be used to manipulate a biological particle and its surrounding liquid, since the electroosmotic (EO) flow can allow for an “on the fly” dynamic flow redirection within a device for further data collection and analysis [19]. Both AC and DC electric potentials can be used to exploit differences in specific biological particle properties, such as electrical charge, size, shape, and polarizability [20–21]. An important fraction of miniaturized EK devices employ a combination of electrophoresis (EP), dielectrophoresis (DEP), and EO flow effects [22], allowing for additional parameters to fine-tune a separation process. Particle characterization and manipulation with EK techniques offer the potential for developing novel separation schemes for the sorting and enrichment of biological particles of interest. Furthermore, it has been shown that nonlinear EK effects, such as EP of the second kind [23–24], are very effective mechanisms for controlling particle migration within microdevices. An advantageous strategy for enhancing nonlinear EK effects on a particle is to use insulator-based EK devices, in which insulating structures distort the electric field distribution generating regions of higher electric field strength within the device [25]. These are simple devices usually made from a single substrate, which makes EK methods at the microscale promising for high-throughput purification applications. Furthermore, a recent development in the field has been the use of a universal design parameter, the electrokinetic equilibrium condition proposed by Cardenas-Benitez et al. [26], which only depends on particle characteristics and can be used to optimize the design of EK separation experiments. The unique particle-dependent parameter *E*_EEC_ can predict the required particle trapping voltage in any insulator-based EK device. A modified version of the *E*_EEC_, called empirical *E*_EEC_ (e*E*_EEC_) can be obtained with a less labor-intensive procedure by employing devices with insulating posts, as proposed by Coll De Peña et al. [25].

EK microscale techniques can be used to manipulate particles across several sizes [27], ranging from proteins [28–30], to viruses [31], cells [32–33], and parasites [34]. Traditionally, these type of studies at the microscale were labeled as DEP methods, although more recently it has been reported that the major phenomena controlling the particles in these systems is the EP of the second kind [25–26]. The use of DEP for protein manipulation via dielectrophoresis chromatography was first reported in 1994 by Washizu et al. [35]. Since then, other studies [36–38] have demonstrated the potential of DEP for macromolecule manipulation. However, EK microscale techniques for protein manipulation are still being developed. The Ros group has reported both the trapping and streaming of protein particles with devices containing insulating posts on the micro- and the nanoscale [29,39–41] as well as the sorting of protein nanocrystals with a streaming insulator-based DEP technique [42–44]. The Swami and Chou groups have studied protein particle enrichment in high-conductivity media employing devices with nanoscale gaps and reported both positive and negative DEPs [28,45–47]. Other studies have employed triangular insulating posts with nanoscale gaps [48], cylindrical insulating structures to enrich BSA particles [49], or diamond-shaped posts to separate PEGylated ribonuclease A from non-PEGylated molecules [50].

The potential advantages of using EK microscale techniques with insulating structures include facilitating the manufacture and high-throughput parallel processing, which makes EK methods key candidates to be used in purifying SPNPs for clinical applications. Besides exploiting size differences, EK microscale techniques can also exploit differences in both electrical charge and dielectric properties of the particles, making them uniquely suited methods to process and purify SPNPs. EK techniques can also be used to differentiate between SPNPs and ASPNPs, since the anisotropic particles, the polar ends of which have different properties, should display different behavior patterns in the microdevices than their single-compartment counterparts. In this work, we present the characterization of SPNPs synthesized via EHD co-jetting in a custom-built system, as well as the application of EK techniques to characterize SPNPs composed of BSA and lysozyme. Two types of particles were synthesized: particles that were homogenous blends of proteins and anisotropic particles. A total of eight distinct types of SPNPs were electrokinetically characterized. The results illustrate that particle composition strongly influences the voltage at which SPNPs will become trapped in an insulator-based EK device. Moreover, homogeneous particles with a higher content of BSA showed lower trapping voltage values. The results also indicate that particle anisotropy plays an important role in determining the trapping voltage, as 50:50 homogeneous SPNPs require a much lower trapping voltage than 50:50 anisotropic SPNPs. All eight particle samples were characterized in terms of their linear and their nonlinear electrophoretic mobility, and in terms of their *E*_EEC_. These measurements expand the possibilities to use *E*_EEC_ to characterize nanoparticles and allow for the accurate prediction of SPNPs behavior in any EK device. The results obtained here shed light on the great potential of insulator-based EK devices to be used for the analysis and purification of protein nanoparticles.

## Results and Discussion

### Synthesis and characterization of synthetic protein nanoparticles

**Figure 1:**
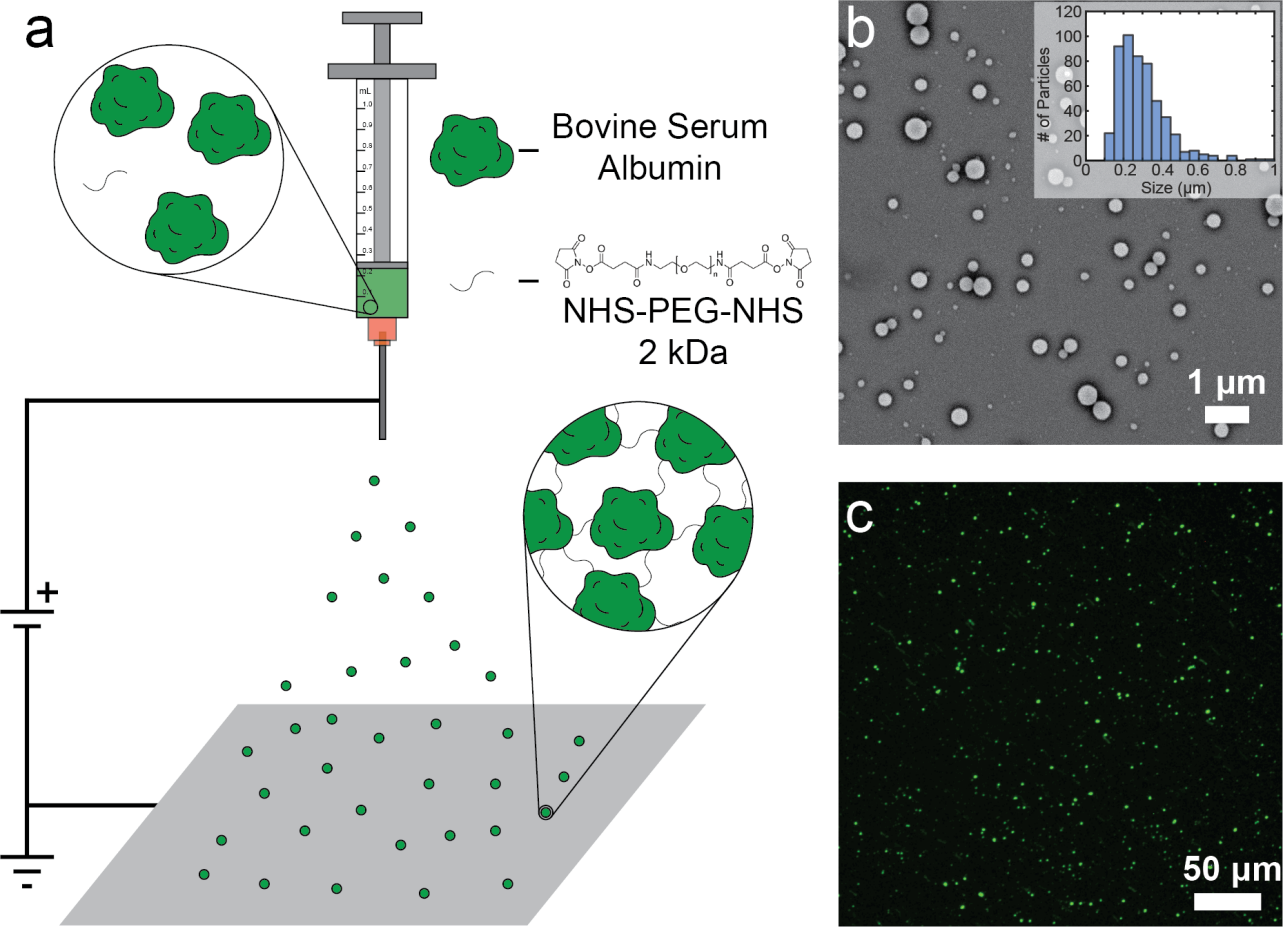
Fabrication process of synthetic protein nanoparticles (SPNPs). (a) A representation of the electrohydrodynamic jetting process, which was used to prepare SPNPs with a single compartment (identical jetting solutions are flown through the needle) as well as two compartments (parallel flow of two different jetting solutions). (b) A scanning electron microscopy (SEM) image demonstrating the morphology of the as-synthesized SPNPs, using SPNPs made from BSA as an example. All SPNPs studied showed indistinguishable morphologies when viewed with SEM. (c) Image of SPNPs loaded with BSA-Alexa 488 to make them visible for characterization via electrokinetic microscale experiments. The fluorescence was confirmed by using a fluorescence microscope.

SPNPs were synthesized using a modification of the well-established EHD co-jetting technique [51–53]. Generally, SPNPs are generated by dissolving a protein of interest and a copolymer of choice into a co-solvent system of water and an organic solvent, such as ethanol or ethylene glycol [16]. The mixture is then ejected through a small-gauge needle towards a collecting surface. A high voltage (kilovolts range) is applied between the needle and the collecting surface, which leads to the formation of a Taylor cone in the solution droplet at the end of the needle. At the tip of the Taylor cone, the fluid jet breaks and the nanoparticles are formed (Figure 1a). EHD co-jetting builds off electrospraying by incorporating a second parallel syringe to create anisotropic, or multicompartmental, structures. With the use of varying device designs and synthetic parameters, a variety of different particle and fiber architectures can be made [54–55].

Bovine serum albumin (BSA) SPNPs were synthesized via the EHD co-jetting method, with BSA as the protein and an oligomer composed of poly(ethylene glycol) (PEG, 2 kDa) terminally bifunctionalized with *N*-hydroxysuccinimide (NHS) esters as the copolymer. The NHS esters react with the lysine groups of BSA and polymerize into insoluble SPNPs. The SPNPs had a spherical shape according to SEM images. We further analyzed the size distribution of the particles using the ImageJ software and found that the particles had an average diameter of 299 ± 128 nm (Figure 1b). The particles were subsequently collected and suspended in media. After allowing the particles to equilibrate for 24 h, the average particle diameter value increased to 356 ± 190 nm according to dynamic light scattering (DLS) measurements. The size distribution curves obtained from SEM and DLS were similar to those previously obtained from EHD jetting [56]. The size difference between the SEM and DLS measurements resulted from the particle solvation state since they swell in water. A swelling behavior of a similar magnitude was observed in both ultrapure H_2_O and in the low-conductivity EK media used in the subsequent experiments.

To test the potential of using EK microfluidics as a high-throughput SPNP purification technique, our particles had to be fluorescently labeled to be visualized with a fluorescence microscope. To accomplish this, we incorporated commercially available Alexa Fluor dyes conjugated to BSA as fluorescent markers at 0.8% (w/w) of total protein into the initial electrospraying solution. After performing the purification steps described previously, we were able to clearly observe particles under typical microscopy conditions (Figure 1c).

### Effect of small size differences and fluorescent dyes

**Figure 2:**
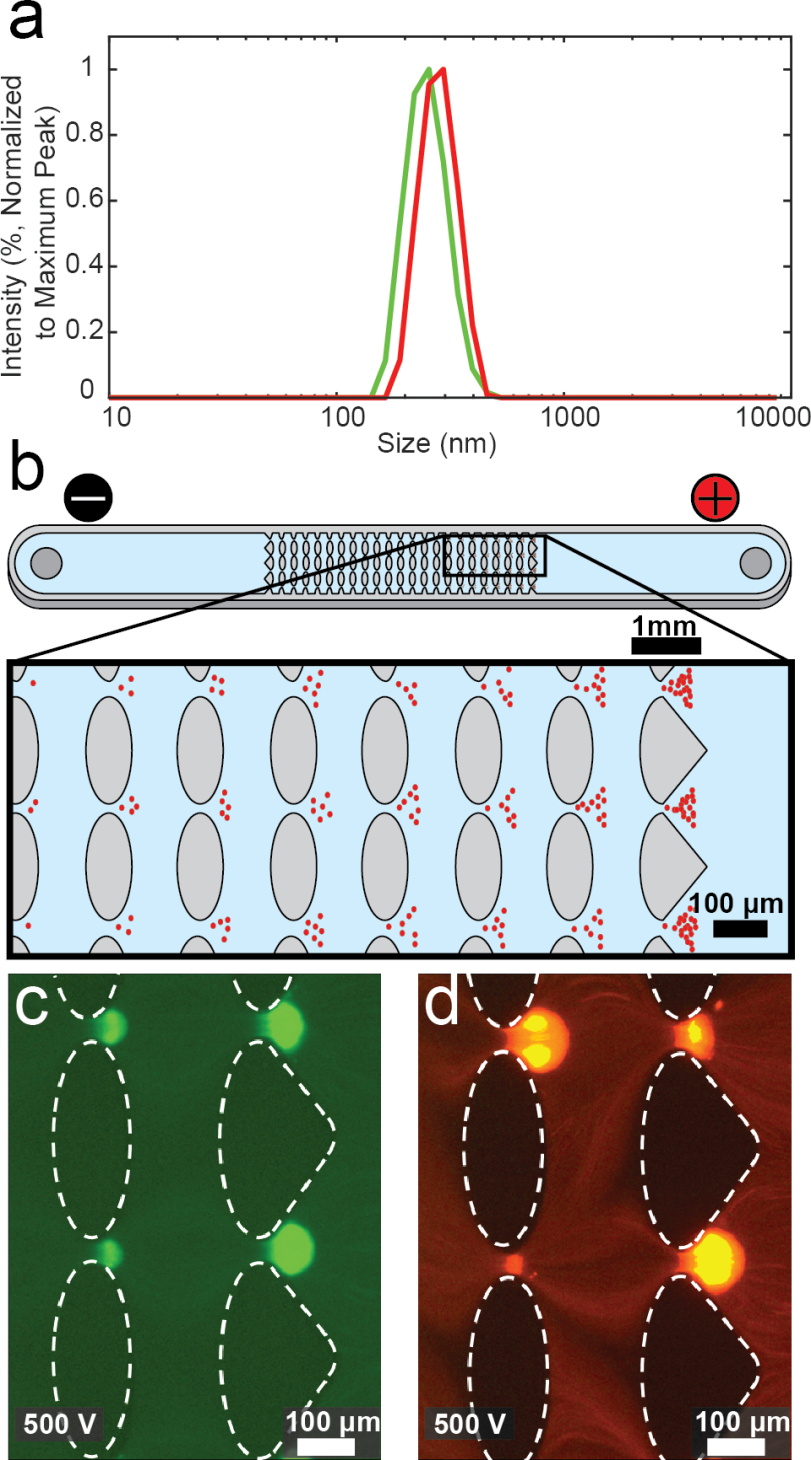
Results illustrating the effect of the fluorescent dye on the SPNP behavior in EK microfluidics. (a) DLS size distribution of BSA SPNPs labeled with Alexa Fluor 488 (green) and Alexa Fluor 555 (red). (b) Schematic representing the EK microfluidic device used and how particles are trapped within the device (inserts). Trapping of SPNP-BSA-488 (c) and of SPNP-BSA-555 (d). Both types of SPNPs were trapped at an applied voltage of 500 V and exhibited a very similar trapping behavior. The flow direction is from the positive to the negative electrode (right to left) in images (b–d).

For potential applications in theragnostics, in which fluorescently labeled particles could be needed, we were first interested in investigating whether the incorporation of different fluorescent dyes in our particles could affect the particle behavior in EK microfluidics. Thus, we synthesized BSA SPNPs loaded with either Alexa Fluor 488 (SPNP-BSA-488) or Alexa Fluor 555 (SPNP-BSA-555) and explored their behavior in an EK device. The resulting particles were fully characterized, and it was found that SPNP-BSA-488 and SPNP-BSA-555 had hydrodynamic diameter values of 373 ± 180 nm and 356 ± 190 nm, respectively (Figure 2a). The zeta potential values (ζ_p_) of the SPNPs were also measured via electrophoretic light scattering in the same suspension medium used in the EK experiments. The obtained results were −20.0 ± 0.6 mV for the SPNP-BSA-488 and −17.4 ± 0.6 mV SPNP-BSA-555.

The EK devices to study these particles were made using microdevice manufacturing techniques as previously described. Standard soft lithography techniques were used to cast polydimethylsiloxane (PDMS) onto molds, and the resulting microdevices were sealed with PDMS-covered glass wafers to ensure all the internal walls had the same zeta potential. These microchannels were designed to include an inlet and an outlet liquid reservoir in which electrodes are placed, and an array of PDMS insulating posts located at the center of the channel (Figure 2b and Figure S1, Supporting Information File 1). The particles are introduced at the inlet reservoir prior to applying an electric potential. In the devices, upon the application of an electric potential the particles will begin migrating in the device towards either the inlet or outlet reservoirs. The particles stop migrating (i.e., become trapped) at a certain voltage at the constrictions between the insulating posts. The voltage at which a particle is trapped is directly related to the properties of the particle (i.e., electrical charge, size, shape, and polarizability). Therefore, every particle will be trapped at a different voltage. These differences in trapping voltage can be used to separate and characterize particles. The trapping voltage can also be used to estimate the *E*_EEC_, which is a parameter that can be used to predict the trapping behavior across any microdevice design, for each particle type. These calculations will be shown later.

SPNP-BSA-488 and SPNP-BSA-555 were both measured in the EK microdevices and both particle types were found to be trapped at the same potential, that is, 500 V (Figure 2c and Figure 2d). This result suggesting that the presence of the dye does not affect the EK response has been reported in the literature in similar EK microdevices [57]. The absence of a difference in trapping voltage even in the presence of different fluorescent dyes shows that the dye molecules themselves have no evident effect on the EK behavior of the particles.

### Electrokinetic response of SPNPs composed of different proteins

EK microfluidics could potentially be used to separate SPNPs of two different proteins based on size and ζ_p_ value. Thus, we next investigated the trapping voltage of SPNPs composed of two commonly used model proteins, BSA and lysozyme. The two proteins have significantly different isoelectric points, 11.35 for lysozyme and 5.4 for BSA. This difference in isoelectric point implies that the two particles would behave differently in an EK device. SPNPs were synthesized using two main proteins: lysozyme (SPNP-Lys-488) or BSA (SPNP-BSA-488). The particles were fully characterized (Table 1). SPNP-Lys-488s had a size distribution and ζ_p_ value similar to those of the BSA SPNPs when measured in the EK buffer (Figure S2, Supporting Information File 1). The small differences in size distribution were mainly due to stochastic variabilities during particle synthesis; however, all the particles had a similar shape and were indistinguishable from each other when visualized using SEM. While surprising, considering the different isoelectric points, the lack of a difference in ζ_p_ value was likely due to the low-conductivity suspension media used. Conversely, when the particles were measured in Dulbecco’s phosphate-buffered saline (DPBS), they had significantly different values for ζ_p_, which were −3.5 ± 0.5 mV and 11.2 ± 0.9 mV for SPNP-BSA-488s and SPNP-Lys-488s, respectively.

**Table 1:** Composition of eight distinct types of PNPs studied. The BSA and lysozyme composition percentage as well as their respective hydrodynamic diameters and zeta potentials measured in EK suspension medium (i.e., the buffer in which subsequent EK characterization experiments were conducted) are listed.

Particle sample	Particle composition	Hydrodynamic diameter (nm)	ζ_p_ (mV)

BSA	100% BSA	356 ± 190	−20.0 ± 0.6
lysozyme	100% lysozyme	277 ± 130	−20.5 ± 2.2
90:10 blend	90% BSA 10% lysozyme	367 ± 201	−20.6 ± 1.0
75:25 blend	75% BSA 25% lysozyme	302 ± 168	−14.6 ± 0.5
50:50 blend	50% BSA 50% lysozyme	360 ± 169	−14.5 ± 0.9
25:75 blend	25% BSA 75% lysozyme	304 ± 100	−22.1 ± 1.9
10:90 blend	10% BSA 90% lysozyme	266 ± 94	−20.3 ± 1.8

SPNP-Lys-488s were then analyzed using an EK device, as previously described, and the particles were trapped at 2300 V. This voltage was significantly different from 500 V, which was the voltage obtained for SPNP-BSA-488s. This result suggests that the two particle types could be easily differentiated via EK microfluidics.

### Electrokinetic response of SPNPs as function of the particle composition

**Figure 3:**
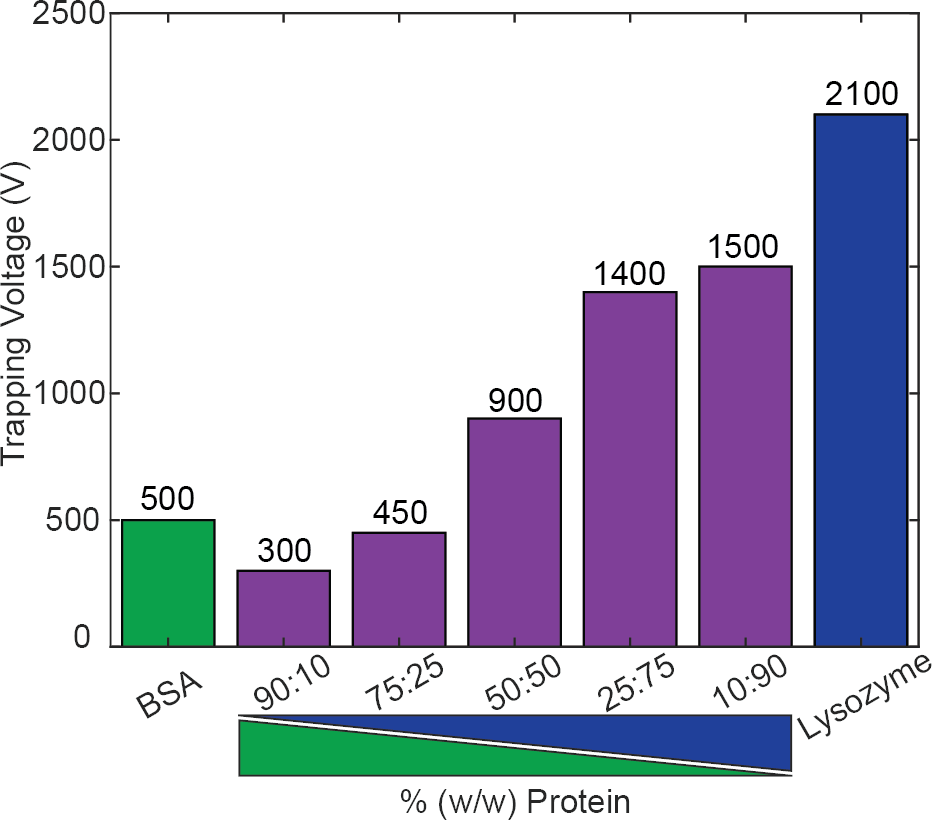
Experimental results of the trapping voltage of seven types of SPNPs composed of BSA, lysozyme, or blends of both proteins. The image at the bottom illustrates how the trapping voltage values vary depending on the SPNP composition. The voltage required to trap the particles increased when the amount of lysozyme also increased. Images of trapped SPNPs are included in Figure S3 (Supporting Information File 1).

SPNPs to be used for therapeutic purposes could be made from a mixture of different proteins in a single particle, with a carrier protein and an active protein (e.g., an enzyme) [6]. To investigate the sensitivity of EK microfluidics to detect differences in blends of two proteins in single particles, SPNPs were synthesized using a variety of ratios of BSA and lysozyme. Particle composition, size, and ζ_p_ value are listed in Table 1. Since DLS measures the particles in their hydrated state (hydrodynamic diameter), it shows the size of particles most similar to the one that they have in the EK devices. These were the sizes that we have reported. For a similar reason, we measured the two parameters for all particles after they were equilibrated in the EK buffer used in the subsequent experiments. The particles were then analyzed using an EK microdevice. Generally, when the amount of lysozyme in the particles was increased, the voltage required to trap the particles also increased (Figure 3). This result shows that EK microfluidics is a promising technique to detect SPNPs that are composed of blends of different proteins. Images depicting the trapping of eight synthesized particle samples are included in Figure S3 (Supporting Information File 1).

### Electrokinetic response of ASPNPs

**Figure 4:**
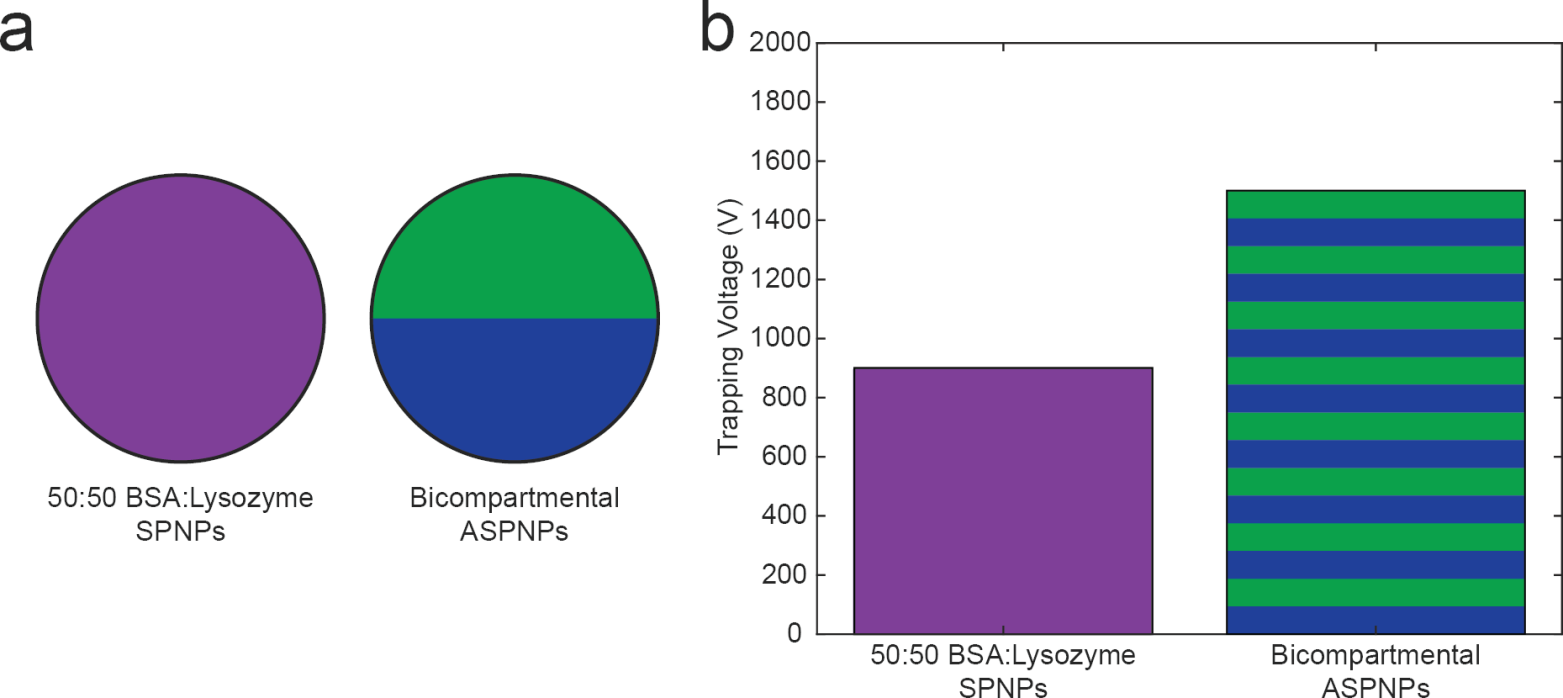
PNP material distribution and trapping voltage characteristics. (a) Schematic representation of SPNPs composed of a homogeneous mixture of BSA and lysozyme, and ASPNPs made in a bicompartmental configuration. b) Graph showing the trapping voltage values that were significantly different between the two types of SPNPs analyzed.

A last interesting point was to determine whether EK microfluidics could differentiate particles based not only on their general composition, but also on local anisotropy. Thus, ASPNPs with one hemisphere composed entirely of BSA and the other one of lysozyme were synthesized. When imaged using SEM, these particles were indistinguishable from their single-compartment counterparts (Figure S4, Supporting Information File 1). The ASPNPs have a size distribution similar to that of the other particles (322 ± 172 nm), and a ζ_p_ value of −14.1 ± 2.7. Note that this would be compositionally equivalent to the 50:50 particles tested earlier (Figure 4a). However, the measured trapping voltage of the ASPNPs was 1500 V. Therefore, there is a difference in trapping voltage between ASPNPs and particles made of a 50:50 (w/w) ratio of BSA and lysozyme, which was 600 V (Figure 4b). This voltage difference is significantly higher than the possible step size of the EK microfluidics, which is in the range between 23 and 50 V [25]. Although the two particle types were identical in material composition, EK microfluidics was able to differentiate the subpopulations purely based on the distribution of matter within each particle. The unique trapping voltage of ASPNPs in comparison to protein-blend SPNPs is notable and it shows that ASPNPs can be potentially sorted using EK microfluidics.

### Estimation of nonlinear electrokinetic parameters

**Figure 5:**
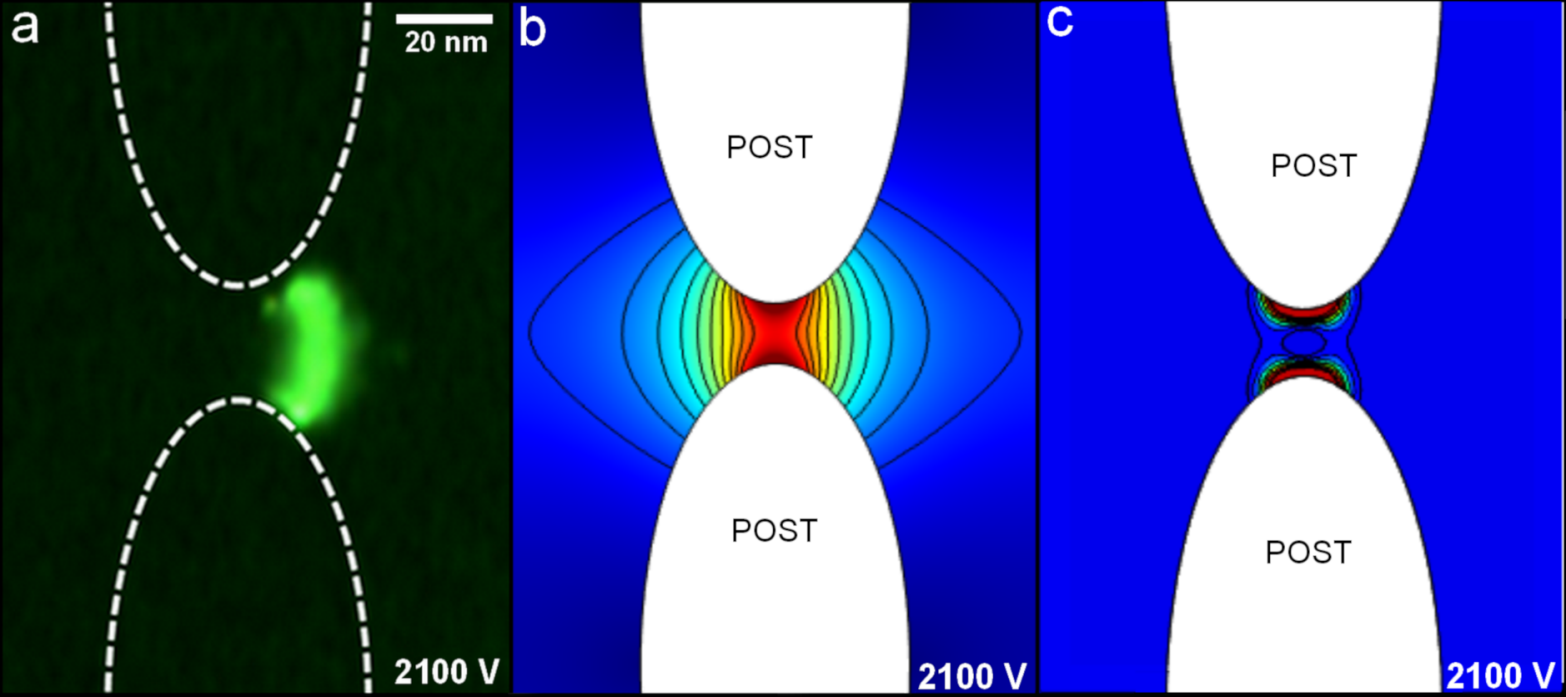
(a) A representative image of trapped lysozyme SPNPs at 2100 V. (b) Plot of the electric field magnitude inside a post constriction with black isoelectric field lines at an applied voltage of 2100 V. (c) Plot of the gradient of the square of the electric field inside a post constriction at an applied voltage of 2100 V.

After the applied voltage necessary to trap each type of SPNP was obtained, it was then possible to derive the electric field magnitude at which each SPNP type would have zero velocity (i.e., *E*_EEC_). The previously developed technique [25] to calculate the e*E*_EEC_ of particles relies on the fact that trapped particles (Figure 5a) become trapped along isoelectric lines (Figure 5b). The effects of DEP depend on the gradient of the square of the electric field (Figure 5c). The isoelectric line with the lowest magnitude in Figure 5b (i.e., the line passing through the midpoint of the constriction) forms a “barrier” through which only particles with an e*E*_EEC_ higher than the electric field magnitude along this line can pass. With this information obtained from the experiments, the e*E*_EEC_ of each SPNP listed in Table 2 was calculated by simulating the electric field within a constriction using the COMSOL Multiphysics software at the trapping voltage of each particle. For the simulations, a conductivity of 21.3 µS·cm^−1^ and a relative permittivity of 78.4 for the suspension medium were used, while the substrate was assumed to be an insulator due to the low conductivity of PDMS [58].

**Table 2:** Electrokinetic properties of eight distinct types of SPNPs studied.

particle sample	EP^(1)^ mobility 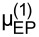 (m^2^·V^−1^·s^−1^)	eEP^(3)^ mobility 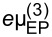 (m^4^·V^−3^·s^−1^)	e*E*_EEC_ (V·m^−1^)

BSA	−1.44 × 10^−8^	−1.15 × 10^−18^	2.31 × 10^5^
lysozyme	−1.45 × 10^−8^	−1.37 × 10^−19^	6.68 × 10^5^
90:10	−1.49 × 10^−8^	−3.17 × 10^−18^	1.38 × 10^5^
75:25	−1.04 × 10^−8^	−1.51 × 10^−18^	2.08 × 10^5^
50:50	−1.05 × 10^−8^	−3.78 × 10^−19^	4.15 × 10^5^
25:75	−1.58 × 10^−8^	−1.43 × 10^−19^	6.46 × 10^5^
10:90	−1.43 × 10^−8^	−1.28 × 10^−19^	6.92 × 10^5^
bicompartmental	−1.01 × 10^−8^	−1.37 × 10^−19^	6.92 × 10^5^

The nonlinear empirical electrophoretic mobility 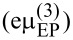 was obtained by employing Equation 1 with e*E*_EEC_ values, since both µ_EO_ and 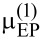 were experimentally obtained a priori through particle image velocimetry and current monitoring measurements [59]. Equation 1 and its detailed derivation is included in the Theoretical approach section. The linear and the nonlinear EP mobility components are essential for the design of EK particle separation experiments. The present report is a pioneer study that considers nonlinear behavior of PNPs. This is still an evolving theory and, as such, more research regarding this topic is required as stated in three recent review articles on protein electrokinetics [30,60–61].

## Conclusion

In summary, we have shown a potential high-throughput method to characterize and manipulate synthetic protein nanoparticles. We synthesized eight distinct types of SPNPs with a variety of characteristics, ranging from different fluorescent dyes and protein compositions to different particle anisotropy (homogenous and anisotropic). Particles were tested in EK microfluidic devices with oval insulating posts, which demonstrated the potential to use EK techniques for the rapid enrichment and characterization of SPNPs. Notably, the results illustrated that by employing insulator-based EK microfluidics, it is possible to differentiate SPNPs by two distinct characteristics, that is, protein composition and anisotropy. The electric potential required to electrokinetically trap and enrich homogenous SPNPs depends on the particle composition (i.e., the relative fractions of BSA and lysozyme). The higher the lysozyme fraction within the SPNPs, the higher the electric potential required. In terms of particle anisotropy, homogenous SPNPs required a much lower electric potential to be electrokinetically trapped than ASPNPs separated into two hemispheres. Furthermore, a complete EK characterization for all eight SPNPs samples was reported, including the zeta potential value of the particles, two electrophoretic mobility types, and the empirical electrokinetic equilibrium condition (e*E*_EEC_) [25]. The latter parameter is an essential component that integrates the main EK phenomena acting on the particles, and it has the potential to be used for the design and optimization of these systems. The e*E*_EEC_ is analogous to an EK signature, that is, this value remains constant for a particular particle type in any insulator-based EK system. Differences in the dielectric properties of particles can be exploited for the design of EK-based separation processes. These results demonstrate that EK microfluidics is a promising technique to be used for the characterization and separation of synthetic protein nanoparticles.

## Theoretical Approach

Depending on the magnitude of the electric field, as particles move through an insulator-based microchannel the predominant forces will ultimately dictate the particle behavior. At lower magnitudes, the predominant force will be linear electrokinetics, EK, which is composed of electro-osmosis (EO) and linear electrophoresis (EP^(1)^). The velocity expressions associated with these components are given by [62]:

[2] $$v_{\text{EO}}=\mu_{\text{EO}}\;E=-\frac{\varepsilon_{\text{m}}\;\zeta_{\text{W}}}{\eta }\;E,$$

[3] $$v_{\text{EP}}^{\left(1\right)}=\mu_{\text{EP}}^{\left(1\right)}\;E=\frac{\varepsilon_{\text{m}}\;\zeta_{\text{P}}}{\eta }\;E,$$

where **v**_EO_ and 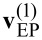 are the electroosmotic and linear electrophoretic velocities, respectively. µ_EO_ and 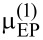 represent the electroosmotic and linear electrophoretic mobility parameters, respectively. Moreover, µ_EO_ depends on the zeta potential of the PDMS (ζ_W_) and 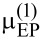 on that of the particle (ζ_p_), while both mobility values depend on the permittivity (ε_m_) and the viscosity (η) of the media.

However, at relatively high electric fields, the effect of nonlinear EK, in particular electrophoresis of the second kind (EP^(3)^), is amplified due to its cubic dependence on **E**, and will therefore dictate particle behavior. This relationship is further developed by Shilov et al. who reported that at higher electric field strength values **v**_EP_ had a nonlinear dependence on **E** [23]. Furthermore, they suggest that **v**_EP_ is an odd function and thus even powers will not be present in the expansion of **v**_EP_. Moreover, given that the voltage decrease across a particle under the conditions of our experiment is larger than the thermal voltage (25 mV), the models presented by the Dukhin group and the Yariv group can also be used to describe the EK behavior observed within our system [24,63–64]. The voltage decrease in this study ranges from 25 to 111 mV, as estimated by the particle radius times the electric field (r_p_·**E**). While the models differ on how the EP^(3)^ mobility is determined, they concur in the definition of the EP^(3)^ velocity and the first two terms in the expansion of the EP velocity:

[4] $$v_{\text{EP}}^{\left(3\right)}=\mu_{\text{EP}}^{\left(3\right)}\;(E\cdot E)\;E,$$

[5] $$v_{\text{EP}}=\mu_{\text{EP}}^{\left(1\right)}\;E+\mu_{\text{EP}}^{\left(3\right)}(E\cdot E)\;E,$$

where similar to 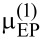, the electrophoretic mobility of the second kind 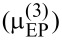 does not depend on the electric field either [63].

Differences in electrical charge of the particles can be enough to elicit a separation via electrophoresis under a linear EK regime. However, the particle size can also be exploited in the presence of EP^(3)^, as it is proportional to the square or the cube of the particle size, depending on which model is used [24,63–64]. In addition, a second nonlinear phenomenon to consider is dielectrophoresis, which is defined as the particle migration due to the polarizability of particles when exposed to a non-homogenous electric field. The DEP velocity is characterized by the following equation:

[6] $$v_{\text{DEP}}=\mu_{\text{DEP}}\;\nabla E^{2},$$

where µ_DEP_ is the DEP mobility. Therefore, summarizing the four phenomena discussed above, the total particle velocity within our system (Figure 2b and Figure S1, Supporting Information File 1) can be estimated by:

[7] $$v_{\text{p}}=v_{\text{EO}}+v_{\text{EP}}^{\left(1\right)}+v_{\text{EP}}^{\left(3\right)}+v_{\text{DEP}}.$$

However, based on estimated values in a recent study [25] it is possible to neglect the effects of **v**_DEP_ for simplification purposes, since **v**_DEP_ had a relatively low contribution to the 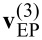 (0.89% to 5.85% for biological cells). Thus, total particle velocity can be represented as:

[8] $$v_{\text{p}}=v_{\text{EO}}+v_{\text{EP}}^{\left(1\right)}+v_{\text{EP}}^{\left(3\right)}=\mu_{\text{EO}}\;E+\mu_{\text{EP}}^{\left(1\right)}\;E+\mu_{\text{EP}}^{\left(3\right)}\;(E\cdot E)\;E.$$

The SPNPs used in this contribution are negatively charged and |ζ_w_| > |ζ_p_| (ζ_w_ = −97.3 mV, which yielded µ_EO_ = 7.58 × 10^−8^ m^2^·V^−1^·s^−1^. The mobility data was measured experimentally with current monitoring [59]). This means that the linear EK and nonlinear electrophoretic motions are in opposite directions. As the particles approach and enter the post array, they will be exposed to areas of a higher electric field, where the opposing forces will be equal in magnitude and will therefore yield an overall particle velocity of zero, causing the particles to become trapped. By setting **v**_p_ = 0 in Equation 8, the electrokinetic equilibrium condition (*E*_EEC_) can be expressed as [25–26]:

[9] $$\mu_{\text{EO}}\;E+\mu_{\text{EP}}^{\left(1\right)}\;E=-\mu_{\text{EP}}^{\left(3\right)}\;(E\cdot E)\;E,$$

[1] $$E_{\text{EEC}}=\sqrt{-\frac{\left(\mu_{\text{EP}}^{\left(1\right)}+\mu_{\text{EO}}\right)}{\mu_{\text{EP}}^{\left(3\right)}}},$$

where Equation 1 is used to estimate the threshold for particle trapping. This particle trapping parameter is a factor of both particle charge and particle size. It can be used to separate particles in a channel by trapping one particle while another particle, with different attributes, is able to effectively move through the system. Moreover, it must be noted that in our system 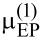 and 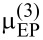 will always be negative since our particles are negative. This makes *E*_EEC_ a real number when µ_EO_ is positive and has a higher magnitude than 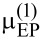, as in our system. In the case of positively charged particles, however, both EP motions will be in the same direction as the EO motion (in theory), causing the particle velocity to increase indefinitely as the applied electric field is increased.

A recent report from our laboratory by Coll De Peña et al. [25] showed a simplified method for determining *E*_EEC_, which requires less experimental work and data analysis. This new parameter is called the empirical electrokinetic equilibrium condition e*E*_EEC_. With this simplified parameter it is possible to predict the condition for the electrokinetic trapping of microorganisms in arrays of insulating posts, as well as to determine the empirical EP of the second-kind mobility 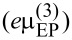 by employing Equation 1 with e*E*_EEC_. The parameters of e*E*_EEC_ and 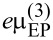 are reported in Table 2 for all the PNPs studied here.

## Experimental

### Nanoparticle synthesis, purification, and characterization

Particles were synthesized using the EHD jetting method. The protein(s) of interest and 2kDa NHS-PEG-NHS copolymer were dissolved at 10% (w/v) and 1% (w/v), respectively, in a 90:10 (ultrapure H_2_O/EtOH) solution. They were subsequently flowed through a syringe at a rate of 0.1 mL·h^−1^, and a voltage was applied between the needle and the collecting surface to produce a Taylor cone. The voltage caused the droplet to be pulled towards the collecting substrate and subsequently the particle broke up into the nanometer-sized spheres. In mid-flight, the solvents rapidly evaporate to form solid nanoparticles. For bicompartmental particles, the needles were placed in a parallel configuration to create a laminar flow at their ends, as described previously, and all other conditions were identical. For the polycondensation reaction, in which the NHS groups in the PEG copolymer react with lysine groups in the proteins, the nanoparticles were placed in a dry oven at 37 °C for 7 days. All SPNP synthesis reagents were purchased from Sigma-Aldrich and Fisher Scientific.

After polycondensation, the particles were collected by scraping them off the collecting surface using a solution of ultrapure H_2_O + 0.01% Tween 20. The collected solution was then sonicated on ice, run through a Falcon 40 μm cell filter (Fisher Scientific), and then centrifuged at 3,200 RCF for 5 min to remove large particles. The resulting supernatant was then centrifuged at 21,130 RCF for 40 min to collect the desired particles.

Particles, in their dry state, were imaged using SEM for shape analysis. To determine their hydrodynamic size distribution after being hydrated, the particles were suspended in ultrapure H_2_O + 0.01% Tween 20, sonicated on ice, and measured with dynamic light scattering (Malvern ZSP ZEN-5600), using standard settings. The average of at least three measurements was reported. Particle zeta potential was measured on the same instrument using a disposable folded capillary cell (DTS1070, Malvern) and using standard settings. Particle concentration was measured using a Pierce BCA assay using BSA standard for the standard curve. Particle fluorescence was confirmed using a Leica DMi8 inverted microscope (Wetzlar, Germany), which was paired with a Leica DFC7000 T camera and the software LASX provided by the manufacturer.

### Microdevice fabrication

Microchannels with oval insulating posts (Figure 2b and Figure S1, Supporting Information File 1) were made from PDMS employing standard soft lithography techniques. To create a device, PDMS (Dow Corning, Midland, MI) was cast onto a negative replica mold made with a silicon wafer (Silicon Inc., Boise, ID) and an SU-8 3050 photoresist (MicroChem, Newton MA). After curing, the PDMS slab was sealed with a PDMS-coated glass wafer using a corona wand (Electro Technic Products, Chicago, IL), creating microchannels where all the internal surfaces are made of PDMS and have the same wall zeta potential (ζ_W_), ensuring consistent EO flow. The microchannels were 10.16 mm long, 0.88 mm wide, 40 µm deep, and contained one inlet and one outlet liquid reservoir.

### Microfluidics experiments

Before placement in the microfluidic device, fully characterized SPNPs were pelleted by centrifugation at 21,130 RCF for 40 min and suspended at a concentration of 2.5 mg·mL^−1^ in ultrapure H_2_O + 0.01% Tween 20. Particles were then diluted to 250 mg·mL^−1^ in the same suspension medium used to fill the device (DI water with added K_2_HPO_4_ to reach pH 6.2 and a conductivity of 21.3 µS·cm^−1^, supplemented with 0.05% Tween 20) and subsequently sonicated to break up particle aggregates. This low-conductivity medium has a limited buffer capacity. A sample of suspended SPNPs (1–5 µL) was injected into a device before the electrodes were inserted and the pressure was equalized. In order to determine the trapping voltage of one particular type of SPNPs, a range of increasing voltage values were tested on a single sample until particle trapping in the form of bands was observed, such as the bands seen in Figure 2a and Figure 2c. Once this approximate trapping voltage was found, a new channel and sample were used to test voltage values at and below the approximate trapping voltage. With the new sample, each time a new voltage was applied, the system was returned to a neutral pressure. Voltage values were decreased by 100 V increments until a smooth trapping band was no longer observed.

## Supporting Information

File 1 Additional characterization information and device schematics.
