# Supplementary material for: Electrokinetic characterization of synthetic protein nanoparticles
Source: Beilstein J Nanotechnol. 2020 Oct 13;11:1556–67. doi: 10.3762/bjnano.11.138 (PMC7590587; doi:10.3762/bjnano.11.138)
Supplement: File 1 — Additional characterization information and device schematics. [file Beilstein_J_Nanotechnol-11-1556-s001.pdf]

BEILSTEIN JOURNAL OF NANOTECHNOLOGY

Supporting Information

for

Electrokinetic characterization of synthetic protein nanoparticles

Daniel F. Quevedo, Cody J. Lentz, Adriana Coll de Peña, Yazmin Hernandez, Nahal Habibi, Rikako Miki, Joerg Lahann and Blanca H. Lapizco-Encinas

Beilstein J. Nanotechnol. **2020,** *11,* 1556–1567. doi:10.3762/bjnano.11.138

Additional characterization information and device schematics

License and Terms: This is a supporting information file under the terms of the Creative Commons Attribution License (https://creativecommons.org/licenses/by/4.0). Please note that the reuse, redistribution and reproduction in particular requires that the authors and source are credited.
The license is subject to the *Beilstein Journal of Nanotechnology* terms and conditions: (https://www.beilstein-journals.org/bjnano)

Electrokinetic microfluidic device

The schematic below (Figure S1) shows the detailed dimensions of the EK device used in this study.

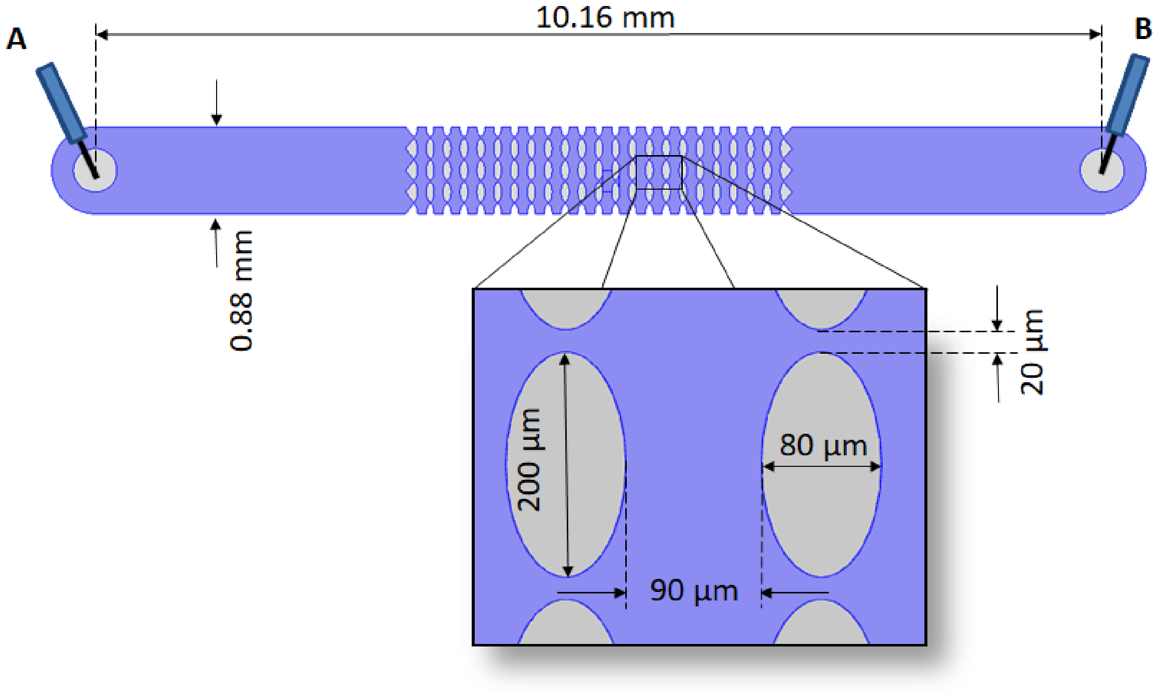

Figure S1: Schematic representation, with dimensions, of the iDEP device used in this study depicting the dimensions of the channels and the insulating posts.

SPNP hydrodynamic diameter size distribution

The graph below depicts the size distribution of all SPNPs studied in this work.

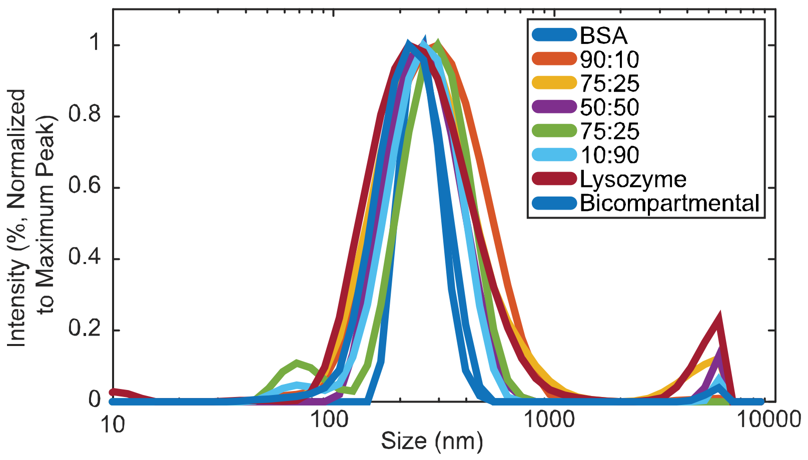

Figure S2: Plot of the size distribution curves of the SPNPs measured with dynamic light scattering.

Trapping of SPNPs in EK microfluidic devices

Figure S3 shows representative images of all SPNPs being trapped in an EK microfluidic device. These images complement the results shown in Figure 4 of the main article.

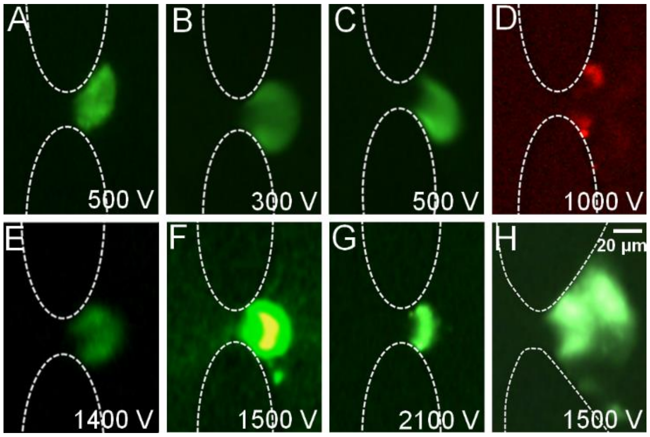

Figure S3: Images depicting the EK trapping of eight types of SPNPs used in this study.

Scanning electron microscopy micrograph of anisotropic SPNPs

The image in Figure S4 shows an example of anisotropic SPNPs with one compartment made of BSA and the other one of lysozyme.

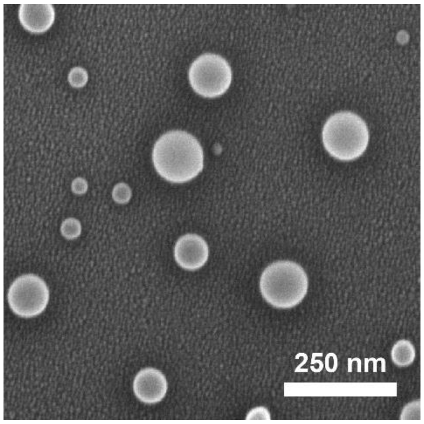

Figure S4: ASPNPs were identical to their single compartment counterparts when viewed using SEM.
